# Prediction model for high glycated hemoglobin concentration among ethnic Chinese in Taiwan

**DOI:** 10.1186/1475-2840-9-59

**Published:** 2010-09-27

**Authors:** Kuo-Liong Chien, Hung-Ju Lin, Bai-Chin Lee, Hsiu-Ching Hsu, Ming-Fong Chen

**Affiliations:** 1 Institute of Preventive Medicine, School of Preventive Medicine, National Taiwan University, Taipei, Taiwan; 2 Department of Internal Medicine, National Taiwan University Hospital, Taipei, Taiwan

## Abstract

**Background:**

This study aimed to construct a prediction model to identify subjects with high glycated hemoglobin (HbA1c) levels by incorporating anthropometric, lifestyle, clinical, and biochemical information in a large cross-sectional ethnic Chinese population in Taiwan from a health checkup center.

**Methods:**

The prediction model was derived from multivariate logistic regression, and we evaluated the performance of the model in identifying the cases with high HbA1c levels (> = 7.0%). In total 17,773 participants (age > = 30 years) were recruited and 323 participants (1.8%) had high HbA1c levels. The study population was divided randomly into two parts, with 80% as the derivation data and 20% as the validation data.

**Results:**

The point-based clinical model, including age (maximal 8 points), sex (1 point), family history (3 points), body mass index (2 points), waist circumference (4 points), and systolic blood pressure (3 points) reached an area under the receiver operating characteristic curve (AUC) of 0.723 (95% confidence interval, 0.677- 0.769) in the validation data. Adding biochemical measures such as triglycerides and HDL cholesterol improved the prediction power (AUC, 0.770 [0.723 - 0.817], *P *= < 0.001 compared with the clinical model). A cutoff point of 7 had a sensitivity of 0.76 to 0.96 and a specificity of 0.39 to 0.63 for the prediction model.

**Conclusions:**

A prediction model was constructed for the prevalent risk of high HbA1c, which could be useful in identifying high risk subjects for diabetes among ethnic Chinese in Taiwan.

## Background

Poor control of type 2 diabetes, presenting as an elevated glycated hemoglobin (HbA1c) level, is associated with macro- and micro-vascular complications among patients with diabetes [1-3]. HbA1c, similar to fasting and post-challenge glucose levels, is a marker for monitoring glucose levels to prevent diabetic complications, such as retinopathy [4]. Furthermore, a high HbA1c level in the general population predicts a further risk of coronary heart disease [5]. Therefore, it is mandatory to construct a prediction model to identify individuals with a high HbA1c level in the general population, despite the low prevalence (1.3%) [6].

A prediction model using anthropometric, lifestyle, clinical and biochemical measures from routine examinations has been developed to identify high-risk individuals for diabetes in cross-sectional [7-10] and prospective cohort studies [11-18]. These models appear to be effective in identifying people with a high risk of diabetes. However, the prediction model for a high HbA1c level is limited [6,19] and there is currently no data available on ethnic Chinese. In this study, a prediction model was constructed and its performance tested in detecting prevalent but unknown levels of high HbA1c in a large, cross-sectional ethnic Chinese population who were recruited from a health checkup program in Taiwan.

## Methods

### Subjects

This cross-sectional study involved 25,452 adult subjects who participated in the health checkup program at the Health Management Center of one tertiary hospital, from January 2003 to December 2006. The sampling strategy for the study population, including inclusion and exclusion criteria, is shown in Additional file 1, Figure S1. After excluding subjects with a history of diabetes with medication, cardiovascular disease, cancer, missing or duplicated data, and age less than 30 years, a total of 17,773 participants were recruited into the study. The study protocol has been described previously [20,21]. Briefly, details of socio-economic status, along with medical and medication histories were collected by questionnaires, and standardized clinical measure procedures were undertaken. The protocol was approved by the hospital's Institutional Research Board. Standardized physical examination procedures, such as anthropometric measures and blood pressure, were also performed [22,23]. Blood pressure was measured in a resting position by trained medical assistants, while body mass index (BMI) was calculated as weight (in kilograms)/square of height (in meters), and waist circumference was measured midline between the low costal margin and superior posterior iliac crest.

### Blood Sampling and Analytic Methods

The procedures for blood sampling and analytic methods have been described in previous studies [22,24]. Briefly, blood samples were collected from each participant after fasting for at least 12 hours. Serum total cholesterol levels were measured using the CHOD-PAP method (Boehringer Mannheim, Germany). HDL cholesterol was measured following precipitation of apolipoprotein B-containing lipoproteins with phosphotungstic acid and magnesium ions (Boehringer Mannheim, Germany). Triglyceride concentrations were measured by the GPO-DAOS method (Wako Co., Japan). The aforementioned lipids were measured using a Hitachi 7450 automated analyzer (Hitachi, Japan). LDL-C concentrations were calculated using the Friedewald formula. CRP was measured by automated nephelometric immunoassay using a Beckman Array instrument (Beckman Array 360 system, Canada). All of the measurements were carried out in a single hospital with a coefficient of variation of 5%. HbA1c levels were measured by automatic high-performance liquid chromatography using a Bio-Rad HbA1c kit (Bio-Rad Diagnostic Group, Hercules, CA, USA) in the central laboratory of the hospital. Standardization using mass spectroscopy and capillary electrophoresis was used, and prepared mixtures of purified HbA1c and HbA0 were used as calibrators [25]. With regards to the cutoff level of HbA1c, abnormally high HbA1c levels were defined as 6.7% according to the sensitivity and specificity of diabetes diagnosis and diabetic retinopathy in one cross-sectional study [26]. In addition, a study by the UK Prospective Diabetes Study Group demonstrated that over 10 years, the mean HbA1c level in their intensive treatment group was 7.0% [1]. Moreover, an HbA1 level of 7.3% or greater is considered the cutoff value for screening diabetes in Pima Indians [27]. Therefore, we set the threshold for abnormally high HbA1c at 7%.

### Statistical analysis

The basic demographic, anthropometric measurements, lifestyle factors, and biochemical measures were described according to a high HbA1c concentration, defined as HbA1c ≥ 7%. The constructed model for HbA1c ≥ 6.5% was similar so that we reported the findings about HbA1c ≥ 7%. Missing waist circumference data in the first year (2003) were imputed with the mean values of waist circumference due to the specific HbA1c status to improve the power of the prediction model. The study population was divided randomly into two parts, with 80% as the derivation data and 20% as the validation data.

Multivariate logistic models were used to predict the risk of a high HbA1c level in the derivation data. First, the Cambridge model [28], including variables of age, BMI, anti-hypertensive medication, family history, and smoking status was used to construct the model [6,19]. Second, an additional anthropometric measurement (waist circumference) and systolic blood pressure were incorporated into the model. History of hypertension medication was excluded due to non-significance in the model. This second prediction model was called the clinical model. Third, important biochemical indicators, including C-reactive protein (CRP), HDL cholesterol, and triglyceride concentrations were added into the model to construct the full biochemical model [29]. Fasting glucose was not included in the model to prevent over-correction by glucose concentration.

Based on results of the multivariate logistic models from the derivation data, two strategies for constructing the prediction model were applied. First, the coefficients for the prediction model from the derivation data were used directly, which is a common strategy in the literature [18,30-32]. By directly calculating the coefficients and individual variables, the individual risk was derived in the validation data. We provided the nomogram using Harrell's method [33]. Second, a point-based chart was constructed from the derivation data according to the strategy suggested by Sullivan and colleagues [34]. This strategy was as follows: continuous variables were organized into meaningful categories and the reference values for each variable were determined. We assigned a 5-year increase in age as the referent risk, and points associated with each of the categories of the risk factors were calculated by comparing with the referent risk. Therefore, an individual's risk was constructed from the validation data by the following formula: *Risk *= 1/[1 + exp(-*βX*)] where β*X *is the sum of the reference risk and the product of the 5-year risk constant and the individual points [34].

Performance of the proposed coefficient-based and point-based prediction models were compared with the Cambridge model [6,19,28]. The area under the receiver operating characteristic curve (AUC) was used to compare the discriminatory capability among the models. A receiver operating characteristic curve is a graph of sensitivity versus 1-specificity (or false-positive rate) for various cut-off definitions of a positive diagnostic test result [35]. Statistical differences in the AUCs were compared using the method of DeLong et al [36].

Furthermore, the goodness-of-fit for all models was assessed based on the Hosmer-Lemeshow test [37]. The global summary statistics included the Brier score [38], twice the forecast-outcome-covariance (a measure of how accurately the forecast corresponds to the outcome, similar to R^2 ^in linear regression) [39], and discrimination (c statistic), which is the same as the AUC [40].

The simple points model was compared with other models using net reclassification improvement (NRI) and integrated discrimination improvement (IDI) statistics [41]. NRI was based on the reclassification tables and was calculated from a sum of differences between the "upward" movement in categories for event subjects and the "downward" movement of non-event subjects [41]. The NRI was presented according to the presumed risk categories of high HbA1c according to quartiles (0.6%, 1.2%, and 2.6%). The IDI was viewed as the difference between improvement in average sensitivity and any potential increase in average "one minus specificity". The statistic was a difference in Yates discrimination slopes between the new and old models [38,42].

All of the statistical tests were two-sided with a type I error of 0.05, and P values < 0.05 were considered statistically significant. Analyses were performed with SAS version 9.1 (SAS Institute, Cary, NC), Stata version 9.1 (Stata Corporation, College Station, Texas) and R http://www.R-project.org.

## Results

### Basic characteristics

Among the study participants, 323 cases (1.8%) had an HbA1c level ≥ 7%. Table 1 shows the basic demographic, clinical, lifestyle, socio-economic status and biochemical measures of the study participants. Participants with higher HbA1c levels were likely to be older, male, have a higher body mass index (BMI), waist circumference, blood pressure, cholesterol, triglycerides, CRP, and white blood cell count, and lower HDL cholesterol level. In addition, participants with higher HbA1c values were likely to take anti-hypertensive medication, have a higher rate of a positive family history of diabetes and current smoking status. The distribution of socio-economic information, such as martial status and job, was similar between participants with and without abnormal HbA1c levels. The distributions of most continuous and categorical variables were consistent in each gender, and there was no differential effect.

**Table 1:** Basic demographic, clinical, lifestyle, and biochemical characteristics of the study population, specified by HbA1c concentration

		HbA1c < 7% n = 17450		HbA1c> = 7% n = 323		
		**Mean**	**SD**	**Mean**	**SD**	**P value**
Age, year		51.0	10.9	56.6	10.2	< .0001
BMI, kg/m^2^		23.8	3.2	25.8	3.7	< .0001
Waist, cm		83.5	9.1	89.6	9.9	< .0001
Systolic BP, mmHg		122.6	16.0	131.6	16.1	< .0001
Diastolic BP, mmHg		73.1	10.5	77.5	10.2	< .0001
Fasting glucose, mg/dL		90.6	10.2	172.0	54.7	< .0001
Postprandial glucose, mg/dL		117.8	48.1	167.7	99.1	< .0001
Total cholesterol, mg/dL		203.7	36.8	220.7	47.3	< .0001
Triglyceride, mg/dL		119.1	75.1	193.0	151.9	< .0001
HDL-cholesterol, mg/dL		44.7	44.1	40.3	8.9	< .0001
LDL-cholesterol, mg/dL		119.6	32.5	134.8	43.5	< .0001
CRP, mg/dL		0.16	0.40	0.31	0.66	< .0001
Uric acid, mg/dL		6.05	1.53	6.03	1.55	0.77
White blood cells		5.43	1.49	6.30	1.76	< .0001
HbA1c, %		5.42	0.37	8.86	1.98	< .0001

		%		%		

Gender	women	44.9		31.6		< .0001
	men	55.2		68.4		
BMI group						< .0001
BMI < 25		67.4		43.0		
25~30		29.1		45.2		
BMI > = 30		3.5		11.8		
Medication history						
Hypertension		12.7		19.2		0.001
Diuretics usage		1.9		4.3		0.001
Lipid lowering		3.2		3.7		0.59
Family history of diabetes						< .0001
	None	71.3		61.3		
	Second relatives	5.4		4.0		
	First relatives	23.3		34.7		
Current smoking	Yes	13.9		20.7		0.0004
Alcohol drinking	Yes	55.6		50.2		0.05
Martial status	Unmarried	11.5		9.5		0.55
	Married	87.7		89.6		
	Separate	0.3		0.6		
	Unknown	0.5		0.3		
Job	Manual work	5.4		5.3		0.23
	Business	24.0		21.7		
	Government, Teacher	21.3		17.3		
	Housework	10.1		12.4		
	No job	4.6		6.5		
	Service	5.8		5.6		
	Student	0.4		0.0		
	Other job	28.6		31.3		

### Constructing the prediction models

The results of the multivariate logistic regression models are listed in Table 2. Hypertension medication was not statistically significant and was excluded in further analyses. Waist circumference and BMI were both associated with abnormal HbA1c levels in the clinical model. Age, family history of diabetes, waist circumference, systolic blood pressure, and biochemical measures, including CRP and triglycerides, were significantly associated with higher HbA1c levels. HDL cholesterol was borderline inversely associated with higher HbA1c.

**Table 2:** Regression coefficients, standard errors and significant levels of various covariates in the two prediction models among the derivation data

	Clinical			Biochemical		
Variable	Estimated parameter	SEM	P	Estimated parameter	SEM	P
Intercept	-12.906	0.775	< .0001	-11.668	0.922	< .0001
Sex, men vs. women	0.351	0.152	0.021	0.174	0.160	0.28
Age, +1 year	0.042	0.006	< .0001	0.043	0.006	< .0001
BMI, +1 kg/m^2^	0.076	0.031	0.014			
Waist, +1 cm	0.024	0.012	0.046	0.036	0.008	< .0001
Family history	0.710	0.138	< .0001	0.724	0.140	< .0001
Smoking history	0.433	0.173	0.012	0.209	0.178	0.24
Systolic blood pressure, +1 mmHg	0.017	0.004	< .0001	0.016	0.004	0.0003
CRP, +1 mg/dL				0.229	0.079	0.004
HDL, +1 mg/dL				-0.018	0.008	0.029
Triglyceride, +1 mg/dL				0.004	0.001	< .0001

**Figure 1:**
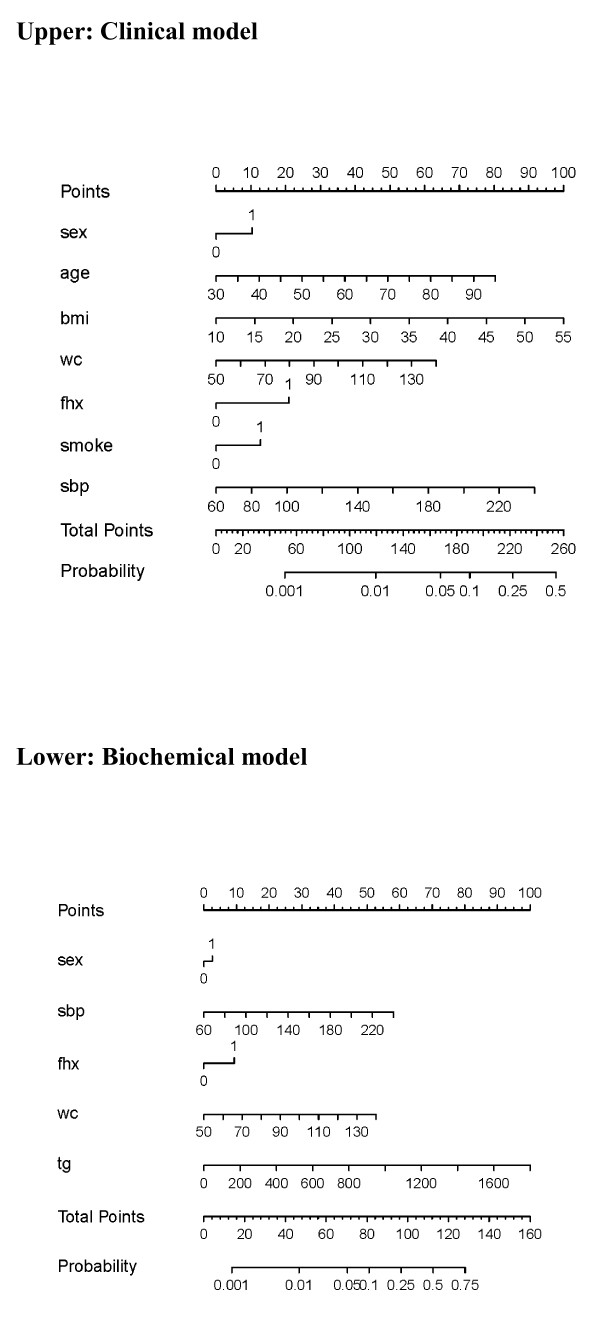
**Nomogram to calculate the probability of high glycated hemoglobin (HbA1c > 7%) using the clinical (upper) and biochemical (lower) models**. In the clinical models, sex (women as 0, men as 1), age, body mass index (BMI), waist circumference (WC), family history of diabetes (FHX), smoking, and systolic blood pressure (SBP) are calculated by reading from the point scale. In the biochemical model, only triglyceride (TG) is calculated. The total point score is then translated into probability of high HbA1c using the bottom scales, including total points and probability. For example, the probability of high HbA1c with a total point score of 170 is then 0.06, according to the two bottom lines. The participants can be classified according to the absolute probabilities accordingly.

Regression coefficient-based and point-based prediction models based on the clinical and biochemical models were developed. A nomogram based on the clinical and biochemical models was constructed (Figure 1). Table 3 shows the point-based clinical model to estimate high HbA1c risk using the points system, derived from the coefficients of the clinical model: age (8 points), sex (1 point), family history (3 points), BMI (2 points), waist circumference (4 points), and systolic blood pressure (3 points). This approach allowed for the manual estimation of the risk of developing a higher HbA1c level for each individual (Table 3). The waist circumference-related point-based biochemical model, additionally including HDL cholesterol (3 points) and triglycerides (2 points), is shown in Additional file 1, Table S1. A cutoff point of 7 had a sensitivity of 0.76 to 0.96 and a specificity of 0.39 to 0.63 for both clinical and biochemical prediction models.

**Table 3:** Simple points system according to the clinical model and the simple points clinical model and absolute risk function for High HbA1c (> = 7%)

**Risk factor**	**Category**	**Point**	**Points total**	**Absolute Risk**
	
Age, yr	30-39	0	0	0.002
	40-49	2	1	0.002
	50-59	4	2	0.002
	60-69	6	3	0.003
	> = 70	8	4	0.004
Sex	Women	0	5	0.004
	Men	1	6	0.005
Family history	No	0	7	0.007
	Yes	3	8	0.008
Current smoker	No	0	9	0.01
	Yes	2	10	0.013
BMI, kg/m2	< 21.1	0	11	0.015
	21.1-22.7	0	12	0.019
	22.8-24.3	1	13	0.023
	24.4-26.2	1	14	0.029
	> = 26.3	2	15	0.035
Waist circumference, cm	<77	0	16	0.043
	77-82.9	0	17	0.053
	83-83.9	1	18	0.064
	84-89.9	1	19	0.078
	> = 90	2	20	0.094
systolic blood pressure, mmHg	<109	0	21	0.114
	109-117.9	0		
	118-125.9	1		
	126-134.9	2		
	> = 135	3		

### Performance measures of the prediction model

**Figure 2:**
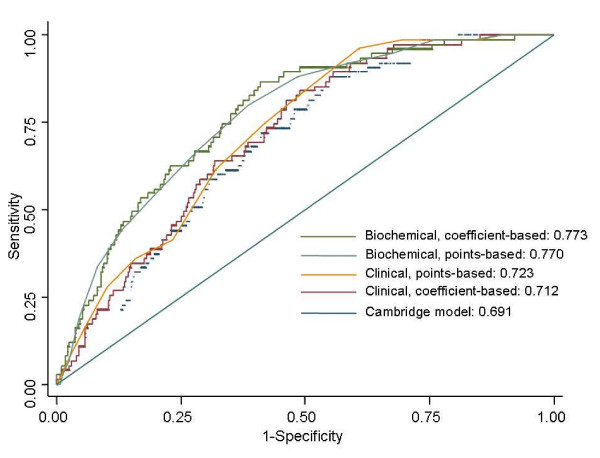
**Areas under the ROC curves for the three prediction models in the validation data**.

The performance of the prediction models, including the Cambridge (Additional file 1, Table S2), coefficient-based and point-based clinical, and biochemical models were compared using different measures (Table 4). The clinical models had a fair discrimination ability with an AUC of 0.712 (95% confidence interval [CI] 0.664-0.760), and 0.723 (95% CI, 0.677-0.769) in the coefficient-based and point-based models, respectively. Adding biochemical measures improved the prediction (AUC of 0.773 [95% CI, 0.726-0.821] and 0.770 [95% CI, 0.723-0.817] for the coefficient-based and point-based models, respectively). Moreover, in the biochemical models, the AUCs were the highest and the Brier scores were the lowest, and they were likely to have a smaller Hosmer-Lemeshow chi-square and high P values, indicating a good calibration ability for a high HbA1c level. Figure 2 shows the AUCs of the various prediction models in the validation data. The Hosmer-Lemeshow chi-square values indicated a goodness-of-fit for these prediction models.

**Table 4:** Summary statistics for different prediction models based on covariates in the Cambridge, clinical, and biochemical model algorithms on the validation data

	Area under ROC curve	Brier score*	2*Forecast Outcome Covariance	Hosmer Lemeshow chi-square**	Hosmer Lemeshow P value**
Cambridge	0.691	0.0219	0.0004	14.6	0.07
Clinical, coefficient-based	0.712	0.0217	0.0007	12.8	0.12
Clinical, points-based	0.723	0.0220	0.0003	18.7	0.03
Biochemical, coefficient-based	0.773	0.0213	0.0013	8.8	0.36
Biochemical, points-based	0.770	0.0219	0.0003	3.8	0.87

*A low Brier score indicated a goodness-of-fit. ** Low Hosmer-Lemeshow chi-square and high P values indicated a goodness-of-fit

A higher area under the ROC area as well as 2*Forecast-outcome-covariance represented better performance. A lower Hosmer-Lemeshow chi-square value represented a goodness-of-fit model.

The performance ability between the various prediction models was tested using NRI and IDI statistics (Table 5). The clinical coefficient-based model was compatible with the Cambridge model (Set 1), and the biochemical models outperformed the Cambridge models due to a significant increase in IDI and NRI values (Set 2). The biochemical coefficient-based model had a better performance than the clinical coefficient-based clinical model by NRI value (Set 3). In addition, the clinical point-based model and clinical coefficient-based model had similar performance measures (Set 4). Finally, the biochemical coefficient-based model outperformed the biochemical point-based model (Set 5), and the clinical and biochemical point-based models had a similar performance (Set 6). The sensitivity, specificity and cutoff values for the clinical and biochemical models were listed in Additional file 1, Table S3. Our findings support that the clinical and biochemical point-based models were excellent models for identifying individuals with a high HbA1c value.

**Table 5:** Comparison of prediction performance by net reclassification improvement (NRI) and integrated difference improvement (IDI) with relative 95% confidence interval (CI) and significance levels

Comparison set	Models	NRI*	95% CI	P value	IDI	95% CI	P value
1	Clinical coefficient-based vs. Cambridge	0.066	-0.054 - 0.186	0.28	0.007	-0.001 - 0.014	0.051
2	Biochemical coefficient-based vs. Cambridge	0.294	0.141 - 0.447	0.0004	0.021	0.004 - 0.037	0.008
3	Biochemical coefficient-based vs. clinical coefficient-based	0.244	0.122 - 0.366	0.0002	0.014	-0.002 - 0.030	0.053
4	Clinical coefficient-based vs. clinical point-based	0.090	-0.022 - 0.203	0.23	0.009	-0.001 - 0.019	0.057
5	Biochemical coefficient-based vs. biochemical point-based	0.362	0.262 - 0.462	0.0003	0.023	0.005 - 0.041	0.006
6	Biochemical point-based vs. clinical point-based	-0.015	-0.139 - 0.109	0.82	0.00003	-0.001 - 0.001	0.49

*Presumed risk categories for NRI according to quartile values (0.6%, 1.2% and 2.6%)

## Discussion

This study confirms and extends the results of a previous study [9] that showed significantly associated risk factors for a high HbA1c concentration in an adult population. Coefficient-based and point-based prediction models for clinical practice were constructed. The biochemical model incorporated several clinical and lifestyle risk factors, as well as biochemical measures, in order to provide a feasible and practical tool for detecting high HbA1c levels. The availability of simple clinical tools to predict the future risk of disease, such as those for predicting coronary heart disease [43-45], can improve predicting the risk of high HbA1c, identify high-risk populations, and enhance preventive strategies.

Thomas and colleagues investigated 7452 45-year-old British adults to compare the predictive power of the Cambridge risk score and body mass index for elevated HbA1c levels [19]. They found that the Cambridge prediction model and body mass index had a similar identifying power for diabetes risk. Park *et al*. collected clinical data from 6567 adults in the European Prospective Investigation of Cancer-Norfolk cohort and showed that the Cambridge model performed well in predicting high HbA1c levels{Park, 2002 [6]}. In several other cross-sectional studies conducted in the US and Europe, prediction models based on clinical information and lifestyle-related factors have appeared to be useful in screening and identifying undiagnosed diabetes cases and high HbA1c levels among various populations [10,28,46,47].

The Cambridge risk model, including age, sex, steroid or antihypertensive medication, smoking, family history, and body mass index, has a specificity of 78% and a sensitivity of 51% to screen cases with HbA1c > = 7.0%[6]. In another screening project for a high HbAc1 level defined as 7.0%, the Cambridge risk model was proven to be a good performance measure [19]. Although different criteria for defining high HbA1c levels for identifying undiagnosed diabetes and screening high risk cases, including 6.5% [9], 7.0% [6] and 7.5% [27], are available, we focused on a cutoff point of 7.0% in this study.

Based on these observations, this study provided a better prediction model than the Cambridge model for predicting a high HbA1c level among ethnic Chinese. We showed that some biochemical measures, especially components related to metabolic syndrome and inflammation such as triglycerides, low HDL cholesterol, and CRP, provide additional information for predicting a high HbA1c level. Aside from fasting glucose and MI, metabolic variables such as high triglycerides and low HDL were found to be strong predictors of type 2 diabetes. These variables also included risk functions developed in other populations [10,48] and were related to a high HbA1c level. Our study did not support a role for LDL cholesterol in identifying a high HbA1c level in the multivariate model.

In our clinical model, a family history of diabetes and systolic blood pressure had significant predictive power for the risk of high HbA1c, and the results imply that these two factors, aside from BMI and waist circumference, should be checked to screen for high HbA1c. Our point-based model clearly showed that family history, BMI, waist circumference, and systolic blood pressure synergistically added to the risk of high HbA1c.

The choice of optimal threshold for defining a high HbA1c level is still inconclusive [26,27,49]. Engelgau and colleagues collected information on diabetic retinopathy and provided the diagnostic threshold of HbA1c as 6.7%. However, Hanson *et al*. used a similar strategy but argued that the best cutoff value should be 7.8%. We used 7.0% as the threshold following previous prediction model results [6,19] and because micro-vascular complications increase appreciably when HbA1c > 7.0%.

The clinical and biochemical models can be implemented easily, and we provide the nomogram for the coefficient-based models. However, calculations are still necessary for the absolute risk probability. Therefore, the point-based models, although with slightly poorer performance than the coefficient-based models, are likely to be implemented. Manual calculation of an individual's risk by summing the points in the point-based models is feasible, such that health professionals can use it in clinical practice. The point-based model using clinical measures may have a useful role in stratifying a population so that those at the highest risk are offered further testing and intervention. A high-risk approach for primary prevention on the risk of diabetes is recommended [50] and the prediction model may be a feasible tool. In addition, multifactorial treatment on risk factors, including weight control, lipids, blood pressure, and glucose level lowering, in patients with type 2 diabetes is a difficult task [51], especially for patients with pre-existing cardiovascular diseases [52]. A high HbA1c level in patients indicates poor glycemic control, so aggressive intervention is necessary for patients with high HbA1c.

To the best of our knowledge, this is the first study on a prediction model specifically developed for the risk of high HbA1c levels among ethnic Chinese. Because of the large sample size, estimates from our prediction models are stable as demonstrated by the internal validation study. Furthermore, the standardization and central laboratory mean the measurements are consistent throughout the study period. The homogeneous study participants provide a reliable estimate for the prediction model coefficients. We consider that these prediction models may be suitable for screening and identifying those at high risk of type 2 diabetes in the Asia-Pacific region.

This study had several limitations. First, the prevalent rate of high HbA1c levels was relatively low (1.8%) so that the predicted risk probability among the general population seems negligible. This low risk, however, may be underestimated. In fact, only 1.3% of participants had high HbA1c levels in the study conducted by Park *et al. *[6]. Therefore, identifying the high-risk population using the prediction model will be a useful tool for further prevention of diabetic complications. Second, the cross-sectional study design made causation difficult. Some anthropometric and lifestyle factors might have been influenced after the onset of diabetes. Our strategy to exclude existing diabetes cases was meant to reduce this reverse causation to as minimal as possible. Finally, we didn't include fasting plasma glucose in the biochemical model due to its high collinearity with HbA1c. In addition, the biochemical model was limited to the population who provided blood samples.

In conclusion, point-based prediction models were constructed to predict the prevalence of high HbA1c levels among ethnic Chinese. These simple clinical tools should help identify high-risk populations and improve prevention and treatment strategies for type 2 diabetes.

## Competing interests

This study has not been published or submitted elsewhere, and no ethical problems or conflicts of interest are declared.

## Authors' contributions

KLC: collecting data, analyzing data, writing the draft, supervising the study; BCL: collecting data; HRL: collecting data; HCH: laboratory data measurements and quality control; MFC: obtaining funding, supervising the study. All authors have read and approved the manuscript.

## Supplementary Material

Additional file 1 **Additional Tables & Figures**. Click here for file
