# Supplementary material for: Prediction model for high glycated hemoglobin concentration among ethnic Chinese in Taiwan
Source: Cardiovasc Diabetol. 2010 Sep 27;9:59. doi: 10.1186/1475-2840-9-59 (PMC2955643; doi:10.1186/1475-2840-9-59)
Supplement: Additional file 1 — Additional Tables & Figures. [file 1475-2840-9-59-S1.DOC]

**Additional Table 1: Simple points system according to the biochemical model and the absolute risk function for High HbA1c (>=7%)**

| Variable | Categories | Points |  | Points total | Absolute Risk |
| --- | --- | --- | --- | --- | --- |
| Age, yr | 30-39 | 0 |  | -3 | 0.001 |
|  | 40-49 | 2 |  | -2 | 0.001 |
|  | 50-59 | 4 |  | -1 | 0.001 |
|  | 60-69 | 6 |  | 0 | 0.002 |
|  | >=70 | 8 |  | 1 | 0.002 |
| Family history | No | 0 |  | 2 | 0.003 |
|  | Yes | 3 |  | 3 | 0.003 |
| Waist, cm | <77 | 0 |  | 4 | 0.004 |
|  | 77-82.9 | 1 |  | 5 | 0.005 |
|  | 83-83.9 | 1 |  | 6 | 0.006 |
|  | 84-89.9 | 2 |  | 7 | 0.008 |
|  | >=90 | 3 |  | 8 | 0.01 |
| SBP, mmHg | <109 | 0 |  | 9 | 0.012 |
|  | 109-117.9 | 0 |  | 10 | 0.015 |
|  | 118-125.9 | 1 |  | 11 | 0.019 |
|  | 126-134.9 | 1 |  | 12 | 0.023 |
|  | >=135 | 2 |  | 13 | 0.028 |
| HDL-C, mg/dL | <36 | 0 |  | 14 | 0.035 |
|  | 36-40.9 | -1 |  | 15 | 0.043 |
|  | 41-44.9 | -1 |  | 16 | 0.053 |
|  | 45-51.9 | -2 |  | 17 | 0.065 |
|  | >=52 | -3 |  | 18 | 0.079 |
| Triglyceride, mg/dL | <65 | 0 |  |  |  |
|  | 65-87.9 | 0 |  |  |  |
|  | 88-115.9 | 0 |  |  |  |
|  | 116-159.9 | 1 |  |  |  |
|  | >=160 | 2 |  |  |  |

Abbreviation: SBP, systolic blood pressure; HDL-C, high density lipoprotein cholesterol;

**Additional Table 2: Regression coefficients, standard errors and significant levels of various covariates in the Cambridge model for the risk of high HbA1c level in the test dataset**

|  | Cambridge | |  |
| --- | --- | --- | --- |
| Variable | Estimated parameter | SEM | P |
| Intercept | -7.789 | 0.380 | <.0001 |
| Sex, men vs. women | 0.495 | 0.150 | 0.001 |
| Age, +1 year | 0.052 | 0.006 | <.0001 |
| BMI, 25-30 vs. <25 kg/m^2^ | 0.750 | 0.142 | <.0001 |
| BMI, >=30 vs. <25 kg/m^2^ | 1.835 | 0.213 | <.0001 |
| HT medication | -0.121 | 0.173 | 0.484 |
| Family history | 0.693 | 0.137 | <.0001 |
| Smoking history | 0.398 | 0.172 | 0.020 |

**Additional Table 3: Sensitivity, specificity and best Youden index of the cutoff values for the clinical and biochemical points-based model.**

| Model | Cutoff value | Sensitivity | Specificity | Youden’s index |
| --- | --- | --- | --- | --- |
| Clinical | 7 | 0.96 | 0.39 | 0.35 |
| Biochemical | 7 | 0.76 | 0.63 | 0.39 |

**Additional Figure 1: Schematic diagram for the study design and participants.**

**
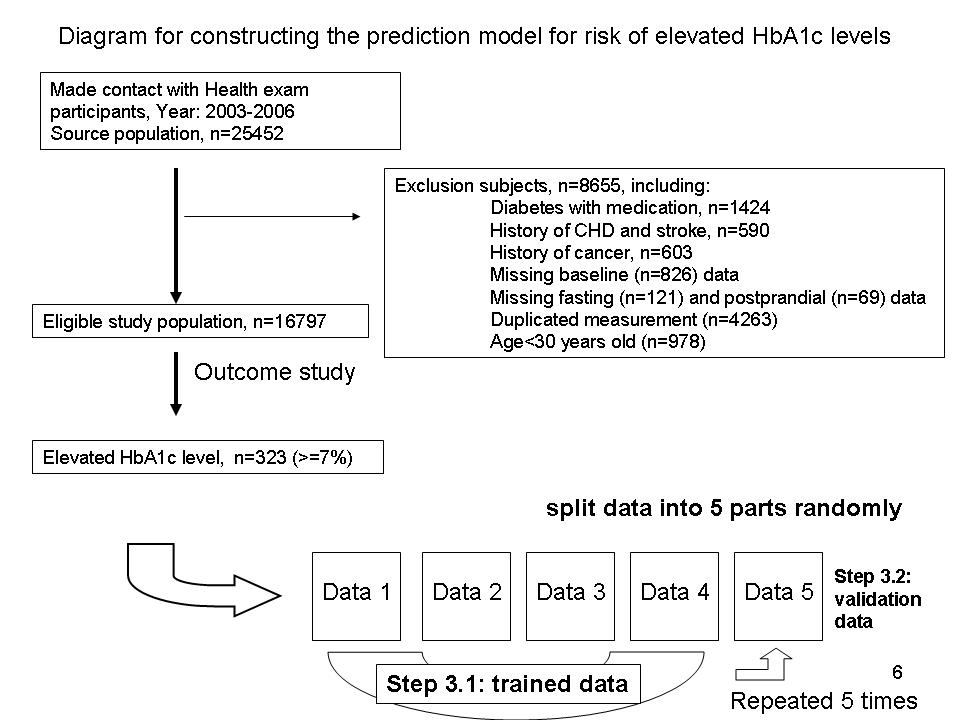
**
